# Hsp90 Orchestrates Stress Response Signaling Governing Fungal Drug Resistance

**DOI:** 10.1371/journal.ppat.1000471

**Published:** 2009-08-28

**Authors:** Leah E. Cowen

**Affiliations:** Department of Molecular Genetics, University of Toronto, Toronto, Ontario, Canada; University of California San Francisco, United States of America

The emergence of drug resistance in microbial pathogens provides a poignant example of an evolutionary process with a profound impact on human health. Fungal drug resistance poses a particular concern given the limited number of clinically useful antifungal drugs and the growing population of immunocompromised individuals vulnerable to life-threatening fungal disease [1]. The efficacy of most antifungal drugs is compromised either by host toxicity, fungistatic rather than fungicidal activity, or by the emergence of drug resistance. Recent studies have revealed that compromising the function of the molecular chaperone Hsp90 can render resistant pathogens more responsive to treatment and can thwart the evolution of fungal drug resistance [2].

## The Molecular Chaperone Hsp90 Regulates the Stability and Function of Diverse Signal Transducers and Modulates the Translation of Genotype to Phenotype

By stabilizing key cellular regulators, Hsp90 can buffer the expression of genetic variation such that it accumulates in a silent state and is exposed when Hsp90 function is compromised, such as by stress [3],[4]. Reducing Hsp90 function reveals new traits in organisms as diverse as flies and plants, with broad implications for evolutionary processes. Cancer provides a poignant example of how Hsp90 can influence somatic evolution on the cellular level. Hsp90 stabilizes mutant oncogenic proteins that are prone to misfolding, thereby enabling malignant transformation [5]. Compromising Hsp90 function can reverse oncogenic traits. Hsp90 has yet another distinct role in fungal evolution: by stabilizing unmutated regulators of cellular signaling, Hsp90 enables stress responses required for survival of drug exposure and for the phenotypic consequences of diverse resistance mutations.

## Hsp90 Enables the Emergence and Maintenance of Resistance to the Azole Antifungals in the Model Yeast *Saccharomyces cerevisiae* and the Leading Fungal Pathogen of Humans, *Candida albicans* (Figure 1)

The azoles are the most widely deployed class of antifungals. They exert fungistatic activity by inhibiting the biosynthesis of ergosterol, the major sterol of fungal cell membranes. Specifically, they inhibit the activity of lanosterol 14α-demethylase (Erg11) in the ergosterol biosynthetic pathway and result in the accumulation of a toxic sterol intermediate that results in cell membrane stress [2]. Compromising Hsp90 blocks the rapid evolution of azole resistance and abrogates resistance that was acquired by diverse mutations [6]. In *S. cerevisiae*, Hsp90's role in azole resistance depends upon the underlying mechanism of resistance. Mechanisms that allow the cell to cope with drug-induced stress, such as loss of function of Erg3, which blocks the accumulation of the toxic sterol that would otherwise accrue when the azoles inhibit Erg11, are critically dependent upon Hsp90 function. Mechanisms that bypass drug toxicity, such as overexpression of drug pumps that efflux the drug from the cell, confer Hsp90-independent resistance. Pharmacological inhibition of Hsp90 reduces resistance of *C. albicans* clinical isolates that evolved resistance in a human host and, importantly, converts the fungistatic azoles into a fungicidal combination [6],[7]. Febrile temperatures reached in humans challenged by infections phenocopy Hsp90 inhibition, reducing fungal drug resistance.

**Figure 1:**
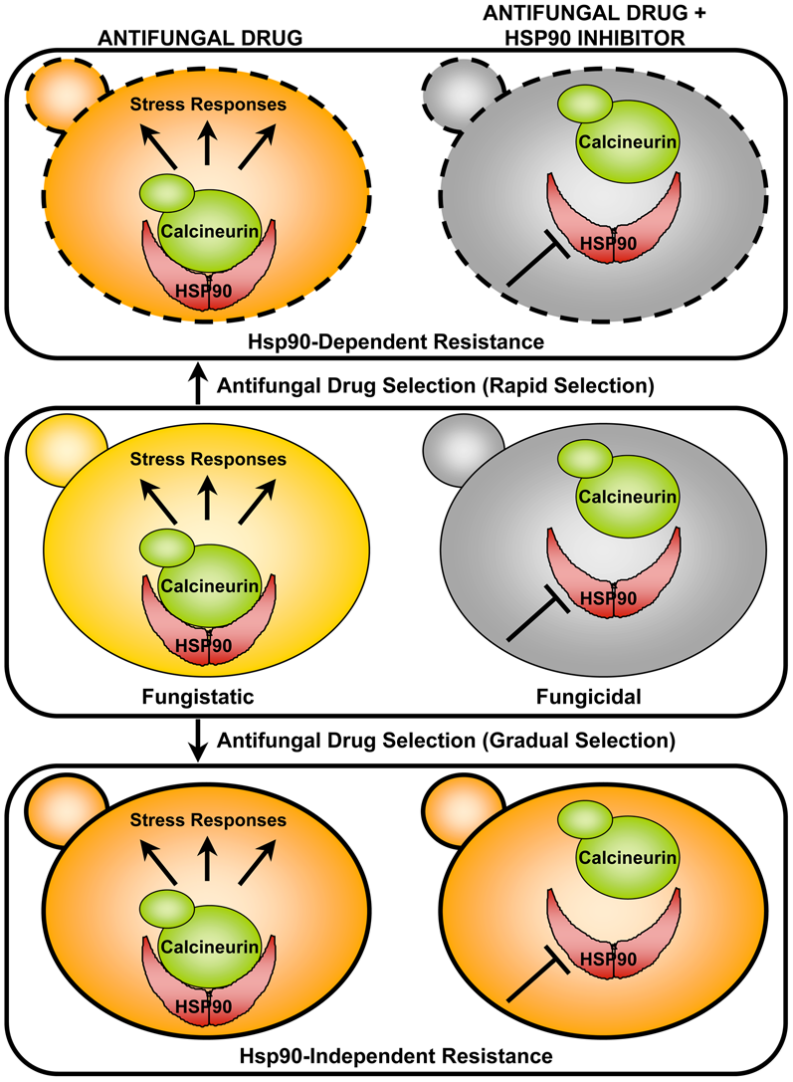
Hsp90's role in fungal drug resistance. Compromising Hsp90 function enhances the activity of fungistatic antifungal drugs, creating fungicidal drug combinations, and can block the evolution of drug resistance. Middle panel, left column indicates that in a wild-type fungal cell (shown in yellow) Hsp90 stabilizes calcineurin, enabling calcineurin-dependent stress responses that are required to survive exposure to fungistatic antifungal drugs (azoles and echinocandins for *C. albicans* and echinocandins for *A. fumigatus*). Middle panel, right column indicates that compromising Hsp90 function abrogates calcineurin-dependent stress responses, rendering fungistatic agents fungicidal (dead fungal cell shown in grey). Top panel, in *S. cerevisiae*, a rapid selection regime favors the emergence of Hsp90-dependent azole resistance (orange fungal cell with dashed perimeter to indicate that resistance is contingent upon Hsp90-mediated stress responses). Bottom panel, in *S. cerevisiae*, a gradual selection regime favors the emergence of Hsp90-independent resistance (orange fungal cell with solid perimeter).

## In *C. albicans* and One of the Most Lethal Moulds, *Aspergillus fumigatus*, Hsp90 Governs Resistance to the Only New Class of Antifungals to Reach the Clinic in Decades, the Echinocandins (Figure 1)

The echinocandins exert fungistatic activity on many fungal species by inhibiting synthesis of (1,3)-β-D-glucan, a critical component of the fungal cell wall, resulting in cell wall stress [1],[2]. Pharmacological or genetic impairment of Hsp90 function reduces echinocandin tolerance of *C. albicans* laboratory strains and resistance of *C. albicans* clinical isolates and creates a fungicidal combination [8]. Pharmacological inhibition of Hsp90 also enhances the activity of echinocandins against *A. fumigatus* [7] and against the emerging pathogen *Aspergillus terreus* [6]. Inhibition of Hsp90 enhances the activity of an azole against *A. fumigatus*, though this effect is contingent on the environmental conditions [7]. Relative to the yeast species discussed above, little is known about the role of Hsp90 in the evolution of drug resistance in filamentous fungi.

## Hsp90 Enables Resistance of Diverse Fungi to Drugs Targeting the Cell Membrane and the Cell Wall via the Client Protein Calcineurin (Figure 1)

Calcineurin is a protein phosphatase that regulates responses to a myriad of stresses in fungal species [9], including a response to azole-induced cell membrane stress in *C. albicans* and a response to echinocandin-induced cell wall stress in both *C. albicans* and *A. fumigatus* [6], [8], [10]–[12]. Hsp90 physically interacts with the catalytic subunit of calcineurin, maintaining it in a stable conformation that is poised for activation [8],[13]. Compromising calcineurin function phenocopies compromising Hsp90 function, reducing fungal drug resistance [1],[2],[8],[9]. In *S. cerevisiae* and *C. albicans*, a key effector of the calcineurin-dependent response to azoles is the transcription factor Crz1. Upon calcineurin activation, Crz1 is dephosphorylated and translocates to the nucleus to activate a stress-responsive transcriptional program [14]. Crz1 plays a partial role in azole tolerance in both yeast species [15],[16] and it also plays a partial role in echinocandin tolerance in *C. albicans* [8], implicating the involvement of other downstream effectors of calcineurin. In *S. cerevisiae*, another downstream effector involved in azole resistance is the integral membrane protein of the endoplasmic reticulum, Hph1, which is dephosphorylated by calcineurin [15]. Since Hsp90 interacts with many other client proteins [17], there may well be many other stress response pathways through which Hsp90 influences drug resistance.

## Hsp90 Provides a Powerful Therapeutic Target for Diverse Fungal Diseases

Inhibiting Hsp90 can enhance the activity of existing antifungals, rendering resistant pathogens more responsive to treatment, and can also block the emergence of drug resistance, creating fungicidal drug combinations. Notably, Hsp90 inhibitors are in advanced phase clinical development as anticancer agents [5]. Combination therapy with Hsp90 inhibitors that are well tolerated in humans and azoles rescues lethal *C. albicans* infections in a tractable and well validated invertebrate host–model system, the wax moth *Galleria mellonella* [7]. Importantly, the efficacies of antifungal therapies in *G. mellonella* larvae correspond well with efficacies in humans and fungal virulence in this model correlates well with virulence in mammalian models of fungal disease [18]. Combination therapy with an Hsp90 inhibitor and an echinocandin rescues larvae from lethal *A. fumigatus* infections [7]. Translation of this novel combination therapy strategy to a mouse model of disseminated *C. albicans* infection is hampered by toxicity associated with inhibiting host Hsp90 in the context of acute fungal infection [7]. However, genetic compromise of *C. albians* Hsp90 expression enhances the therapeutic efficacy of an azole and an echinocandin in a mouse model of disseminated candidiasis, providing genetic proof-of-principle for combination therapy [7],[8]. Further emphasizing the promise of targeting fungal Hsp90, a recombinant antibody against *C. albicans* Hsp90 increased fungal clearance and reduced mortality in combination with amphotericin B in a clinical study [19], though the mechanism by which the antibody mediates these effects remains enigmatic.

Hsp90 is poised to influence diverse facets of fungal biology as a consequence of its function in regulating the activity of a myriad of signal transducers. In *C. albicans*, Hsp90 governs cellular circuitry required not only for drug resistance but also for a key developmental transition from yeast to filamentous growth that is required for virulence [20]. This morphogenetic transition is normally regulated by environmental cues, such as exposure to serum, coupled with elevated temperature that is required to relieve Hsp90-mediated repression of the morphogenetic program. Compromising Hsp90 induces a transition from yeast to filamentous growth by activating Ras1-protein kinase A signaling. Genetic depletion of *C. albicans* Hsp90 results in complete clearance of an infection in a mouse model of disseminated disease [20]. This is consistent with Hsp90's essentiality and its role in morphogenesis, given that morphogenetic flexibility is required for virulence and that compromising Hsp90 drives filamentous growth. Independent of the mechanism, this reinforces the prospect for targeting Hsp90 in fungal pathogens as a powerful therapeutic strategy. Hsp90 inhibitors may provide an even broader therapeutic paradigm for infectious disease. Hsp90 inhibitors possess potent anti-malarial activity, thus extending their spectrum of activity to the parasite *Plasmodium falciparum* [21]. With Hsp90's capacity to sense temperature and orchestrate cellular signaling that governs drug resistance and developmental transitions, it provides an Achilles' heel for diverse pathogens. The challenge ahead lies in developing selective pharmacological agents capable of distinguishing between Hsp90 chaperone machineries of the pathogen and the host.
